# A History of Methamphetamine Use Disorder in People with HIV Is Associated with Altered Functional Response to Risky Choice

**DOI:** 10.3390/v18030369

**Published:** 2026-03-17

**Authors:** Joseph P. Happer, Susan F. Tapert, Igor Grant, Amanda Bischoff-Grethe, on behalf of the Translational Methamphetamine AIDS Research Center (TMARC) Group

**Affiliations:** Department of Psychiatry, University of California San Diego, 9500 Gilman Drive, MC 0738 La Jolla, San Diego, CA 92093, USA; stapert@health.ucsd.edu (S.F.T.); igrant@health.ucsd.edu (I.G.); agrethe@health.ucsd.edu (A.B.-G.)

**Keywords:** HIV, methamphetamine, decision-making, risky behavior, fMRI

## Abstract

Methamphetamine (METH) use is highly prevalent among people with HIV (PWH) and those at risk and may contribute to overall worse health outcomes. Poorer health-related problems may be mediated by METH enhancing risky decision-making among PWH. While both METH and HIV are known to have overlapping and deleterious effects on the frontostriatal neural circuitry essential for decision-making, few studies have examined their combined effects. Eighty-eight participants stratified by HIV and a history of METH use disorder completed a risky decision-making paradigm, which involved choosing among safe (20¢) and risky (40¢/80¢ win or loss) choices, during blood-oxygen level-dependent functional magnetic resonance imaging (fMRI). Linear mixed-effects models were used to assess voxelwise differences in group and choice constrained to the anterior cingulate cortex (ACC), insula, and striatum. Despite similar choice behavior across groups, PWH and a history of METH use disorder had greater activation of the ACC and caudate than either condition alone (i.e., HIV+/METH− and HIV−/METH+), which was similar to seronegative, non-using controls. Within the ACC in particular, these differences may have been driven by safe choices. A longer estimated duration of HIV infection was associated with greater ACC activation to risky choices for PWH regardless of METH use history. These findings suggest that PWH and a history of METH use disorder may exhibit compensatory activation of regions associated with decision-making in the context of rewards and that the effects of HIV and past METH use might not be additive.

## 1. Introduction

A variety of substances of abuse, such as alcohol, stimulants, and opioids, are known to increase the frequency of high-risk behaviors associated with HIV exposure and transmission [1,2,3,4,5]. Methamphetamine (METH) use in particular is highly prevalent among people with HIV (PWH) and those at risk [5,6,7,8] and has been termed a “double epidemic” [7]. METH use among PWH has been associated with poorer health outcomes [3,9,10], likely due to poor medication adherence [10,11] and delayed HIV treatment [12], which may contribute to higher plasma viral loads [9,10,13]. These health-related problems may be due, in part, to METH use enhancing risky decision-making among PWH and those at risk [3,4,5]. Notably, both METH use and HIV are known to have deleterious effects on overlapping brain structure [14,15,16] and function [16,17,18,19,20,21], including on the frontostriatal neural circuitry essential for decision-making. However, few studies have examined the combined impact of METH use and HIV on the neural substrates of risky decision-making despite some studies suggesting their interaction having long-lasting, neurotoxic effects on the brain [3,22,23,24,25].

Risky decision-making can be conceptualized as selecting an outcome from a range of possibilities where the potential for a large gain or loss is prioritized over a choice with a smaller but certain benefit [26,27,28]. Decision-making in risky situations is a complex task requiring integration of multiple functions including reward processing, cognitive control, and response optimization [26]. Extensive neuroimaging studies indicate that these processes are subserved by a frontostriatal distribution of brain regions [26]. Within this network, the insula has been heavily implicated as a node crucial for detecting and processing rewarding and behaviorally salient stimuli [29,30,31,32] while also contributing to the affective response to these stimuli [29,33,34,35]. As part of the dorsal striatum, the caudate nucleus has been demonstrated to be involved in decision-making, particularly as it relates to learning and integrating the outcomes of choices and their reward consequences in support of goal-directed behavior [36]. This contrasts with the ventral striatum, which plays a prominent role in reward processing [30,32] and is involved in the development of addiction and substance use disorders [37]. Top-down regulation and implementation of cognitive control, particularly to override automatic responses in the context of conflicting options, is effectuated by the anterior cingulate cortex (ACC) [31,38,39,40,41,42]. The ACC has widespread anatomical and functional connections with other regions involved in motor planning, attention, and inhibitory control [31,43,44,45,46]. Additionally, more rostral portions of the ACC have been associated with affective engagement of the limbic system in response to emotional and reward-related input [33,34,35], which may help guide behavior via cognitive–affective integration.

As individual conditions, both METH [16,18,47,48,49,50,51,52] and HIV [20,53,54] are known to impact the frontostriatal circuitry underlying decision-making in risky situations. Studies of individuals with a history of METH use have generally reported overall hypoactivation of the ACC during risky decision-making and decision-making more broadly [55,56,57,58], which may reflect alterations in top-down implementation of cognitive control. Attenuated insula and striatal activation have similarly been observed in anticipation of both gains and losses [18,50,57]. Moreover, alterations in insula response to safe and risky decisions have been used to predict METH relapse [59,60] as well as transition into problematic use [61]. Together, these findings indicate adaptations in reward processing and cognitive control circuitry in individuals with a history of METH use that may contribute to problematic use.

In contrast, PWH have frequently demonstrated hyperactivation of frontostriatal circuitry as a function of cognitive demand, particularly in areas necessary for cognitive control such as the ACC and striatum [20,54,62,63,64]. For example, anticipation of riskier choices was shown to elicit greater activation within these regions compared to safer choices for PWH, while people living without HIV (PWoH) demonstrated no such sensitivity [20]. In the absence of behavioral differences, this pattern of activity has been interpreted as compensatory due to decreased neural efficiency following neuronal injury [65] that could stem from chronic inflammatory damage related to HIV infection [22,23]. In contrast, areas related to reward processing including the ventral striatum and insula exhibited decreased activation in anticipation of safer reward outcomes but increased activation for riskier choices in PWH compared to people without [20,62,63]. This may reflect alterations in the value and salience attributed to riskier options. Overall, PWH exhibit potential dysfunction in reward processing with compensatory and greater activation of executive function regions in order to maintain similar behavioral performance.

While previous neuroimaging studies have demonstrated that both HIV [20,53,54] and METH [16,18,47,48,49,50,51,52] independently alter activation of the frontostriatal circuitry subserving decision-making in the context of risk, no studies to date have examined these conditions in combination despite the potential for long-lasting, synergistic neurotoxic effects [3,17,19,22,23,24,25,66]. METH use may exacerbate HIV-associated neuronal damage, leading to more severe and enduring neurocognitive dysfunction even when abstinent [24], which may contribute to continued personal and social risk-taking behaviors associated with poorer health outcomes and HIV transmission. Therefore, the aim of the present study was to examine whether a history of METH use disorder could modulate brain response to risky decision-making in PWH. To do so, we used the Risky Gains Task (RGT) [20,49,52,55], which reliably activates frontostriatal regions including the ACC, striatum, and insula. In line with previous studies of the RGT, we anticipated that overall individuals with a history of METH use may exhibit more risky choices. In comparison to healthy comparison individuals, we hypothesized that both PWH and METH use history would exhibit attenuated activation of the insula in anticipation of making a risky choice, reflecting alterations in reward valuation. If the effects of METH use history and HIV were additive, PWH and a history of METH would show further attenuation in the insula and ventral striatum. In contrast, we anticipated seeing increased activation within cognitive control areas such as the ACC and dorsal striatum in PWH compared to healthy controls while individuals with a history of METH should demonstrate reduced activity. Additive effects of both METH use and HIV could be reflected in reduced activation of these regions, particularly during riskier choices. Finally, we hypothesized that alterations in neural responses during risky choice would be associated with poorer clinical measures in both PWH and individuals with a history of METH use.

## 2. Materials and Methods

### 2.1. Ethics Statement

All study procedures were approved by the Institutional Review Board of the University of California, San Diego. Participants provided written, informed consent prior to enrollment and again prior to scanning. Participants were compensated for their time and effort.

### 2.2. Participants

A total of 172 participants were recruited and completed the Risky Gains Task (described below) during the functional magnetic resonance imaging (fMRI) session as part of a larger National Institutes of Health-funded study conducted through the Translational Methamphetamine AIDS Research Center between 2008 and 2018. Participants were distributed across four groups: PWH with no history of METH use disorder (HIV+/METH−, *n* = 44), PWoH with a history of METH use disorder (HIV−/METH+, *n* = 35), PWH with a history of METH use disorder (HIV+/METH+, *n* = 37), and PWoH and no history of METH use disorder (HIV−/METH−, *n* = 56). A small subset of HIV+/METH− and HIV−/METH− participants was included in a previous analysis [20]. HIV status was confirmed by a MedMira Multiplo rapid test (MedMira Inc., Halifax, NS, Canada). All participants were seronegative for Hepatitis C virus (HCV) as determined by the MedMira Multiplo rapid test. Current CD4 T lymphocyte counts (cells/mL) were determined by flow cytometry at a Clinical Laboratory Improvement Amendments (CLIA), or equivalent, certified medical center laboratory. HIV RNA levels were measured in plasma by reverse transcriptase PCR (Roche Amplicor, v. 1.5, lower limit of quantitation 50 copies/mL). CD4 nadir was obtained by self-report, with confirmation by documented prior measurements in a subset of individuals. Participants were also queried about their current antiretroviral treatment (ART) use including their current regimen and total duration of ART use.

All METH+ participants met diagnostic (DSM-IV) [67] criteria for lifetime amphetamine dependence including abuse or dependence in the past 18 months as determined by the Composite International Diagnostic Interview (CIDI) [68]. METH− participants did not meet criteria for lifetime or current methamphetamine abuse or dependence. Exclusionary criteria for all groups were DSM-IV criteria for alcohol dependence in the past year, other substance dependence in the last 5 years, other substance abuse (e.g., cocaine, opioids) within the last year, and remote (i.e., >5 years) but significant history of alcohol or other drug dependence. Given the high comorbidity of alcohol and marijuana abuse and marijuana dependence with methamphetamine use, METH+ individuals with such histories were not excluded. Given the high rates of mood disorders among both PWH [69,70,71] and substance use disorders [72], participants were not excluded for a current or a history of depression or anxiety diagnoses.

Participants also were excluded for a positive urine toxicology screen or Breathalyzer for illicit drugs (other than marijuana due to its long-lasting detectability) or recent drinking on the day of scan; MRI contraindication; lifetime history of schizophrenia or other psychotic disorder; previous cerebrovascular events as determined by comprehensive neurological exam; head injury with loss of consciousness >30 min or neurologic complications; demyelinating diseases; or seizure disorder. For this analysis, participants were also required to be within the 25–60 age range. Participants were recruited from the San Diego area via flyers and advertisements at community events and drug dependence treatment programs.

### 2.3. Measures

As part of a comprehensive neuropsychological battery [73], participants completed the Wide Range Achievement Test-4 (WRAT-4) as a measure of premorbid intelligence and quality of education [74]. Performance on the neuropsychological battery was summarized using the Global Deficit Score (GDS) [75]. As mood symptoms, particularly depression, are common among many PWH [69,70,71], participants completed the Beck Depression Inventory (BDI-II) [76]. As impulsivity may influence decision-making in risky situations, trait impulsivity was assessed with the Barratt Impulsiveness Scale (BIS) [77,78]. Furthermore, since risky-decision making may be related to sensation-seeking and may represent a common pathophysiology [79], we chose to measure it using the Kalichman Sexual Sensation Seeking Scale (KS4) [80], which assesses personality characteristics and high-risk sexual behavior.

### 2.4. Imaging Procedures

#### 2.4.1. Risky Gains Task

Participants completed the Risky Gains Task, [20,49,52], which consisted of 96 trials with the goal of earning as much money as possible over the course of the task. For each trial, participants were given the option of selecting three possible consecutive outcomes: 20¢, 40¢, or 80¢, with each option appearing on the screen for 1 s in ascending order. The participant had the option to collect the displayed outcome by pressing the button, thereby ending the current trial. Participants were told that 20¢ was the safe option, and they were guaranteed to receive that amount, while 40¢ and 80¢ were risky options in which they could potentially win or lose that amount. Thus, while more money could be gained by waiting until 40¢ or 80¢ options appeared, there was also the risk of losing money. Losses were immediate, and no further button presses could be made. Participants were given no information about the probabilities of winning or losing, but the number of loss trials was set so that choosing the same option on each trial would earn the same final payment. All trials lasted 3.5 s regardless of outcome. Participants received visual and auditory feedback as soon as an option was chosen or a loss was incurred, and the visual feedback remained on the screen until the end of the trial. As an example of a potential loss trial, if a participant was planning for the 80¢ option but was punished at 40¢, they would not be able to press the button to collect 40¢ or gamble to potentially receive 80¢. Punishment occurred on a trial only if a participant did not respond to the previous options. For example, if the 80¢ option was scheduled to be punished but the participant chose 40¢, the participant still won 40¢. Three different trial types were presented in a pseudo-randomized order: non-punished (20, 40, or 80; *n* = 54); punished 40 (*n* = 24); and punished 80 (*n* = 18). Six null trials that required no button response were included as an implicit baseline for neuroimaging purposes.

#### 2.4.2. Image Acquisition

Functional images were acquired in bottom-up interleaved axial slices using T2* weighted echo planar imaging (EPI). Images were acquired on one of two scanners: a 3T GE Discovery MR 750 (Milwaukee, WI, USA) (252 volumes TR/TE = 2 s/30 ms, flip angle = 90°, 64 × 64 matrix, 40 axial slices, 3.75 × 3.75 × 3.0 mm voxels) or a 3T GE Signa HDx (Milwaukee, WI, USA) (252 volumes, TR/TE = 2 s/30 ms, flip angle = 90°, 64 × 64 matrix, 40 3.0 mm (2.6 mm + 0.3 mm gap) axial slices, 3.5 × 3.5 mm voxels). High-resolution T1-weighted fast spoiled gradient echo anatomical images (MR 750: TR/TE = 8.1 ms/3.17 ms, flip angle = 8°, 256 × 256 matrix, 172 sagittal slices, 1 × 1 × 1 mm voxels; Signa HDx: TR/TE = 7.77 ms/2.97 ms, flip angle = 8°, 256 × 256 matrix, 172 sagittal slices, 0.97 × 0.97 × 1 mm voxels) were acquired to permit subsequent activation localization and spatial normalization. Gradient echo field-maps were also acquired to permit compensation for geometric distortions caused by magnetic field inhomogeneity (MR 750: TR = 1 s, TE = 3.7/5.5 ms, flip angle = 60°, 64 × 64 matrix, 160 axial slices, 3.75 × 3.75 × 3 mm voxels; Signa HDx: TR = 1 s, TE = 3.5/5.5 ms, flip angle = 60°, 64 × 64 matrix, 160 3.0 mm (2.6 mm + 0.3 mm gap) axial slices, 3.5 × 3.5 mm voxels). Stimuli were projected onto a screen at the participants’ feet and viewed with the aid of a mirror attached to the 8-channel head coil.

### 2.5. Image Preprocessing

Functional images were preprocessed using Analysis of Functional NeuroImages (AFNI) [81] and FSL [82]. EPIs were slice-time corrected, motion-corrected, and aligned to high-resolution anatomical images using AFNI’s align_epi_anat.py [83]. Movement parameters were visually inspected for extensive motion exceeding 3 mm. Time points with isolated head movements not corrected by coregistration were censored. T1-weighted images were skull-stripped using FreeSurfer’s mri_watershed [84] and registered to the MNI-152 atlas using affine transform followed by nonlinear refinement via FSL’s FLIRT and FNIRT [85,86]. Functional data were aligned to standard space, resampled to 3 mm isotropic voxels, and smoothed to a 6 mm FWHM using AFNI’s 3dBlurToFWHM.

For each participant, AFNI’s 3dDeconvolve was used to determine activation related to the Risky Gains Task. Decision phase regressors were created such that they started at trial onset and ended either when the participant responded or was punished. Five regressors were defined: (1) 20¢, (2) win 40¢, (3) win 80¢, (4) lose 40¢, and (5) lose 80¢. The baseline comprised all other time-points not accounted for by the initial regressors. Additionally, six nuisance motion-related regressors (three translational and three rotational) and a 3rd-order Lagrange polynomial, which accounted for slow signal drift, were included in the baseline. To maximize the signal-to-noise ratio and boost the number of trials available for analysis of 80 choices, a general linear test of win 80 and lose 80 was computed. As choice behavior was the main interest of this study, we opted to combine both 80 win and loss together, as this is a clear choice for the largest reward. In contrast, the same could not be said for combining win 40 and lose 40 because if a participant lost at 40 there was no possibility of choosing the 80 option as trials ended immediately on scheduled losses. Thus, the primary regressors of interest for later analysis were defined in relation to their choice behavior: 20¢ choice (20 win); 40¢ choice (40 win); and 80¢ choice (general linear test of win 80¢ and lose 80¢). Brain activation was operationally defined as percent signal change relative to baseline. Following deconvolution, the beta regressors of interest were converted to percent signal change.

### 2.6. Data Analysis

#### 2.6.1. Demographics

Group-level statistical analyses were performed in R (version 4.3.2) [87] using an HIV × METH model for two-way ANOVAs for continuous variables (e.g., age, education) and logistic regressions for categorical variables (e.g., sex, ethnicity).

#### 2.6.2. Behavioral Analysis

To examine risk-taking behavior, the relative frequency of each choice (20¢, 40¢, 80¢) was investigated. Group-level statistical analyses were performed in R (version 4.3.2) [87] using a linear mixed-effects (LME) model, from R’s nlme package (version 3.1-164) [88]. Choice behavior was modeled as HIV × METH × Choice (20¢, 40¢, 80¢), with Choice as a within-subject factor. Post hoc analyses were performed using R’s emmeans (version 1.8.9) to compute pairwise comparisons [89] while the *p*-values were adjusted using the False Discovery Rate (FDR) [90] method, and standardized effect sizes were reported.

#### 2.6.3. Regions of Interest

Regions of interest (ROIs) were derived from the Harvard-Oxford atlas. The Risky Gains Task (RGT) has been shown to reliably elicit activation of frontostriatal regions including the ACC, striatum, and insula [20,49,52,55]. Therefore, three bilateral ROIs were defined based on these a priori hypotheses and established activation patterns of the RGT: an insula ROI, which contained the insula in its entirety, a striatum ROI that included the caudate, putamen, and nucleus accumbens, and an ACC ROI. These ROIs were used as search regions for all group-level fMRI analyses.

#### 2.6.4. Neuroimaging Analysis

Only participants who made a minimum of 5 choices for each of the possible outcomes were included to ensure adequate signal-to-noise for the fMRI BOLD analysis. Group-level statistical analyses were performed using the nlme package in R to assess differences in blood oxygen level-dependent (BOLD) response. Data were analyzed using an HIV × METH × Choice (20¢, 40¢, 80¢) + Age linear mixed-effects approach. Age was included as a potential covariate to control for the wide participant age range. For all analyses, subject was nested within scanner and treated as a random effect, with HIV, METH, Choice, and Age as fixed effects. Intrinsic smoothness was estimated using the spatial autocorrelation function (acf) option in AFNI’s 3dFWHMx. Minimum cluster sizes were calculated with AFNI’s 3dClustSim in order to guard against false positives. For ROI analyses, a peak voxel of *p* < 0.01 with a cluster threshold of α < 0.0167 was required for significance. This approach employs non-Gaussian models and spatial autocorrelation functions and is more robust than traditional methods [91]. Based on this approach, minimum cluster sizes to be considered significant for each of the ROIs were as follows: 487 μL (18 contiguous voxels) for the ACC, 405 μL (15 contiguous voxels) for the insula, and 381 μL (14 contiguous voxels) for the striatum. An exploratory whole brain analysis examined HIV × METH × Choice activation across the whole brain (peak voxel *p* < 0.001, cluster threshold of α < 0.05, minimum cluster size 351 μL [13 contiguous voxels]) and is presented in the supplement (Table S1). As with the behavioral data, R’s emmeans was used for post hoc analyses of significant clusters, and standardized effect sizes (ESs) were reported.

#### 2.6.5. Primary Robust Regression Analyses

Within-group Huber robust regressions were conducted in R to examine the relationship of clinical variables related to METH use history (age of first use, days since last use, METH use density (total quantity/total days)) within participants with METH use histories, and to HIV infection (illness duration in years, current CD4, nadir CD4) within PWH. Overall trait impulsivity (BIS Total Score) and depressive symptoms (BDI-II) at the time of the MRI scan were also examined for participants with a history of METH use. Measures were log-transformed and z-scored prior to regression. Individual regressions were performed against the mean percent signal change for each choice. Significant clusters were determined within regions of interest using AFNI’s 3dClustSim for small volume correction with a peak voxel of *p* < 0.01. Results were Bonferroni corrected for the number of measures applied to each group and three ROIs, (HIV: α < 0.0056; METH: α < 0.0033). The intersection of significant clusters was then identified between the task-based analysis and the regression analysis. Since the clusters were considered significant within their respective analyses, the resulting overlap can likewise be considered significant [92].

#### 2.6.6. Data Visualization and Presentation

Figures were created with a combination of R packages including ggplot [93], ggsignif [94], and magick [95], while tables were created using gtsummary [96].

## 3. Results

### 3.1. Participant Characteristics

Twenty participants were excluded for age: 10 were older than 60 (3HIV+/METH−; 7 HIV−/METH−) and 10 were younger than 25 (3 HIV+/METH−; 1 HIV−/METH+; 1 HIV+/METH+; 5 HIV−/METH−). Nine were excluded for excessive motion (3 HIV+/METH−; 3 HIV−/METH+; 1 HIV+/METH+; 2 HIV−/METH−), 52 were excluded for only making safe choices (14 HIV+/METH−; 10 HIV−/METH+; 10 HIV+/METH+; 18 HIV−/METH−), and 3 were excluded for having a positive urine toxicology screen the day of the fMRI session (1 HIV+/METH−; 1 HIV+/METH+; 1 HIV−/METH−). A Fisher’s exact test indicated that participants were not excluded based on group for only making safe choices (*p* = 0.78). This left a final sample of 88 participants (20 HIV+/METH−; 21 HIV−/METH+; 24 HIV+/METH+; 23 HIV−/METH−).

Groups did not differ on age, education, ethnicity, and WRAT-4 standard score (Table 1). There was a main effect of HIV for sex, χ^2^ = 10.0, *p* = 0.002, Cramer’s *V* = 0.32, with more males with HIV than without HIV. Individuals with a history of METH use had overall higher global deficit scores, *F*(1,83) = 4.4, *p* = 0.04, η_p_^2^ = 0.05. However, average GDS scores were still below the recommended 0.5 cutoff score for impairment ([mean ± SD] METH−: 0.26 ± 0.3; METH+: 0.40 ± 0.4) [75]. An additional logistic linear regression based on METH history confirmed that there was no difference in the number of individuals who were above the GDS impairment cutoff, χ^2^ = 1.6, *p* > 0.2, Cramer’s *V* = 0.11. A main effect of METH history was observed for BDI scores at the time of the MRI scan, where people with a history of METH use had higher scores than those without a history, *F*(1,80) = 7.0, *p* = 0.010, η_p_^2^ = 0.08. A main effect of METH history was observed for BIS total scores, where participants with a history of METH had higher scores than those without, *F*(1,80) = 16.2, *p* < 0.001, η_p_^2^ = 0.17. On the subscales of the KS4, no differences in non-sexual sensation-seeking were found. However, individuals with a history of METH use self-reported greater sexual sensation-seeking, *F*(1,81) = 10.7, *p* = 0.002, η_p_^2^ = 0.11, and sexual compulsivity *F*(1,80) = 5.5, *p* = 0.02, η_p_^2^ = 0.06. There was also a strong trend for PWH to self-report greater sexual sensation-seeking, *F*(1,81) = 3.7, *p* = 0.06, η_p_^2^ = 0.06.

Participants with a history of METH use disorder (HIV−/METH+, HIV+/METH+) did not differ on their self-reported age of first use (η_p_^2^ = 0.00), total days used (η_p_^2^ = 0.04), days since last use (η_p_^2^ = 0.00), total estimated quantity of METH used (η_p_^2^ = 0.07), use density (η_p_^2^ = 0.05), or primary method of use (Cramer’s *V* = 0.10). Fifteen METH+ participants met criteria for lifetime cannabis abuse or dependence (8 HIV−/METH+; 7 HIV+/METH+) while 29 met criteria for lifetime alcohol abuse or dependence (16 HIV−/METH+; 13 HIV+/METH+). No significant differences were detected between METH+ groups (cannabis: χ^2^ = 0.6, *p* > 0.4, Cramer’s *V* = 0.0; alcohol: χ^2^ = 3.3, *p* = 0.07, Cramer’s *V* = 0.23).

PWH (HIV+/METH−, HIV+/METH+) did not differ in terms of infection duration (η_p_^2^ = 0.03), percentage virally suppressed (Cramer’s *V* = 0.0), current CD4 count (η_p_^2^ = 0.00), reported nadir CD4 count (η_p_^2^ = 0.05), percentage on ART (Cramer’s *V* = 0.00), current ART regimen (Cramer’s *V* = 0.0), or cumulative duration of ART (η_p_^2^ = 0.00).

A sensitivity analysis was additionally completed for the participant demographic and clinical characteristics for participants who were excluded due to only making safe choices. No substantive differences were observed (see Supplementary Materials Table S2).

### 3.2. Behavioral Analyses

On the Risky Gains Task, there was a main effect of Choice, *F*(2168) = 43.6, *p* < 0.0001, η_p_^2^ = 0.34 (Figure S1). Post hoc *t*-tests indicated that participants primarily made safe (20¢) choices, which were selected more than both risky choices (40¢: *t*(168) = 14.0, *p* < 0.0001, ES = 2.1; 80¢: *t*(168) = 17.2, *p* < 0.0001, ES = 2.6). Participants similarly chose the less risky option (40¢) more than the riskiest (80¢) choice, *t*(168) = 3.3, *p* = 0.001, ES = 0.5. Choice behavior was not modulated as a function of HIV or METH status nor as an interaction between them (all *p*s > 0.2). To ensure that exclusionary criteria based on choice behavior did not influence any findings, additional analyses of the behavioral data were completed with all participants included (see Supplementary Materials). No differences in the patterns of choice behavior were found.

### 3.3. Region-of-Interest Analyses

#### 3.3.1. Main Effect of Choice

Significant clusters displaying a main effect of Choice across all participants were identified with centers of mass within the left caudate nucleus and bilateral insula (Table 2; Figure S2). Post hoc analyses indicated a general pattern across clusters for the safe choice (20¢) to elicit a decreased BOLD response relative to both risky choices (40¢, 80¢).

#### 3.3.2. Main Effect of HIV

A main effect of HIV status was detected within the left insula and right putamen (Table 2; Figure S3) where, regardless of METH use history, PWH exhibited overall greater BOLD signal relative to PWoH.

#### 3.3.3. Main Effect of METH

There was a main effect of METH status within the bilateral insula (Table 2; Figure S4). Participants with a history of METH use displayed a greater BOLD response during the RGT compared to those without a history of METH use.

#### 3.3.4. HIV × METH Interaction

**Figure 1:**
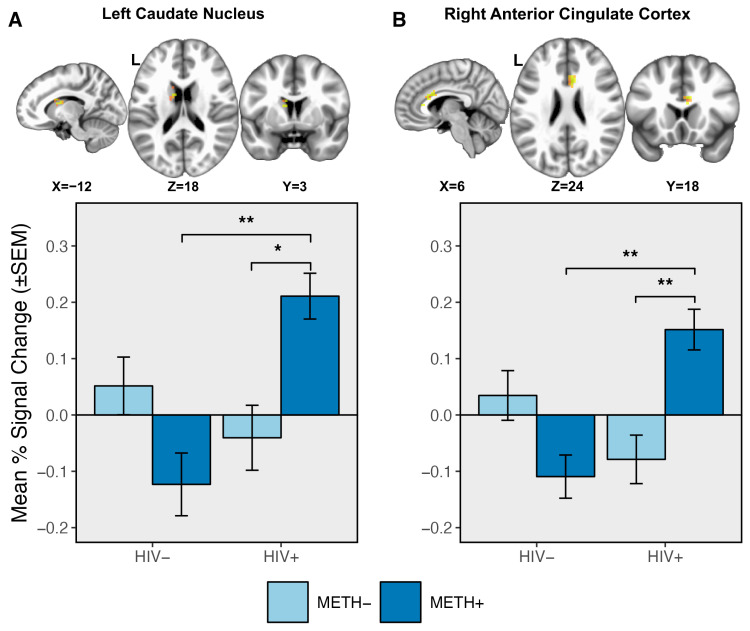
Bar plots showing a significant HIV × METH interaction within two clusters. Within the left caudate nucleus (**A**) and right (**B**) and left (not shown) anterior cingulate cortex, participants with HIV who also had a history of METH use disorder (HIV+/METH+) exhibited overall greater BOLD response compared to participants with HIV but no history of METH use (HIV+/METH−). The BOLD response was similarly greater in the HIV+/METH+ group relative to the HIV−/METH+ group. ** *p* < 0.01; * *p* < 0.05.

Clusters displaying an HIV × METH interaction were detected within the bilateral ACC and left caudate nucleus (Table 2, Figure 1). In all clusters, post hoc analyses revealed that within PWH, METH+ participants displayed an overall greater BOLD response relative to METH−. No differences were detected in PWoH as a function of METH use history (all *p*s > 0.1). Within METH+ participants, PWH had an overall increased BOLD response compared to PWoH, while no differences were observed in METH− participants as a function of HIV status (all *ps* > 0.1).

#### 3.3.5. HIV × METH × Choice Interaction

**Figure 2:**
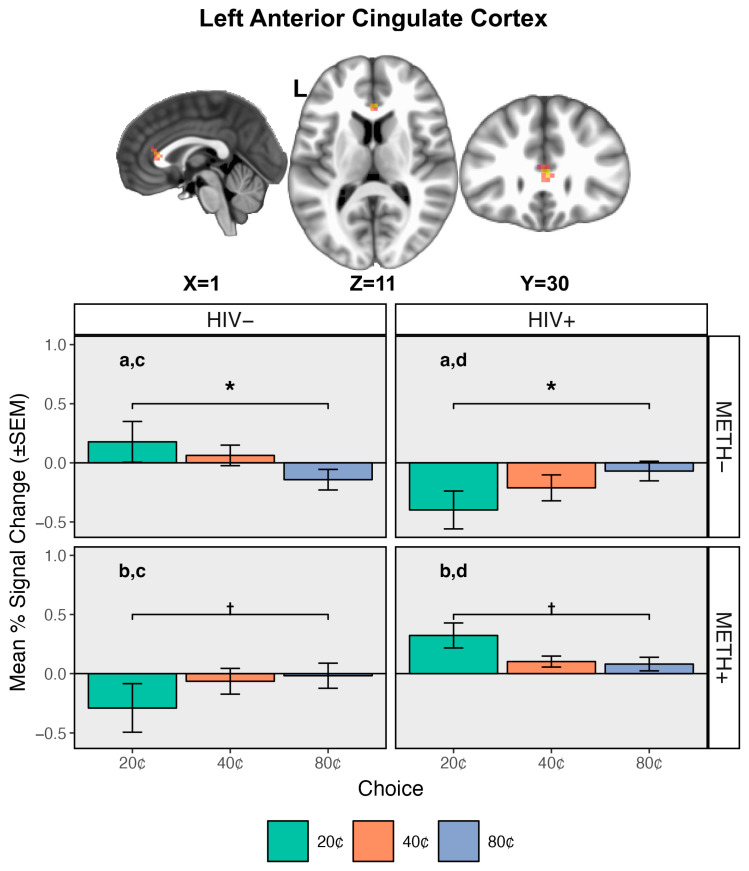
Bar plot showing a significant HIV × METH × Choice interaction in the left anterior cingulate cortex. BOLD responses specifically to safe choices (20¢) varied as a function of both HIV and METH status. For participants without a history of METH use disorder (METH−; comparison a), people with HIV (HIV+) had decreased BOLD compared to those without HIV (HIV−). For people with a history of METH use disorder (METH+; comparison b), HIV+ participants had increased BOLD compared to HIV− participants. For the HIV− group, METH+ participants had decreased BOLD compared to METH− (comparison c). Within the HIV+ group, METH+ participants demonstrated greater BOLD compared to METH− participants (comparison d). Across groups, the safe choice (20¢) generally demonstrated the greatest change in BOLD relative to the riskiest choice (80¢), though this was only at trend level for the METH+ groups. All letter comparisons were significant at *p* < 0.05. * *p* < 0.05; † < 0.07.

A cluster exhibiting an HIV × METH × Choice interaction was found in the left ACC (Table 2, Figure 2). BOLD responses specifically in anticipation of safe choices (20¢) varied as a function of both HIV and METH status. For PWH, METH+ participants displayed greater BOLD relative to METH− participants during safe choices. For PWoH, METH+ participants exhibited decreased BOLD to safe choices compared to METH−. Within those who did not have a history of METH use (METH−), PWH exhibited decreased BOLD compared to PWoH. The opposite pattern was observed in those with a history of METH use (METH+), meaning that PWH exhibited greater BOLD than PWoH in anticipation of safe choices. In sum, individuals who were controls (HIV−/METH−) or had dual diagnoses (HIV+/METH+) exhibited increased BOLD during safe choices (20¢), while individuals with only an HIV diagnosis (HIV+/METH−) or only a history of METH use (HIV−/METH+) had decreased BOLD. No differences in BOLD response during risky decisions (40¢, 80¢) were observed as a function of HIV or METH status.

Additionally, across groups, the safe choice (20¢) generally elicited the largest change in BOLD response relative to the riskiest choice (80¢), though this was only at the trend level for the HIV−/METH+ (*p* = 0.057) and HIV+/METH+ (*p* = 0.067) groups.

#### 3.3.6. Sensitivity Analyses

Given both ART exposure and regimen and viral suppression may individually impact neurocognitive and neuroimaging outcomes [54,97,98], sensitivity analyses were conducted. Separate analyses included (1) only PWH who were on ART and (2) only PWH who were virally suppressed at the time of the experiment (see Supplementary Materials Tables S3 and S4). However, no major differences in findings were observed.

#### 3.3.7. Associations with Clinical Variables

**Figure 3:**
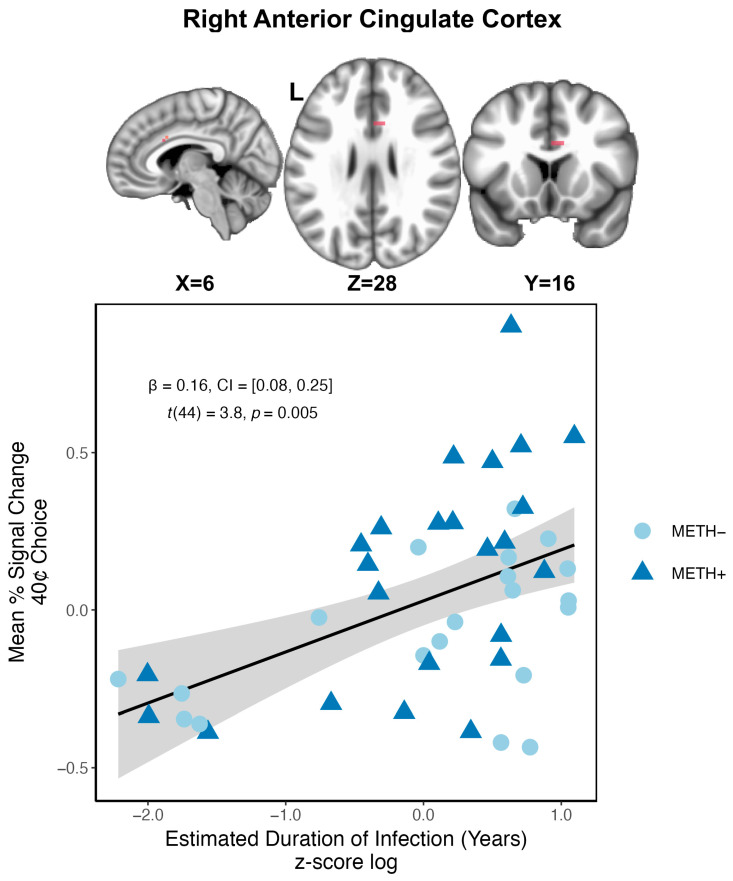
Cluster within the right anterior cingulate with significant relationship to HIV-related clinical variable. Longer estimated duration of HIV infection was associated with greater BOLD response to 40¢ (risky) choices for people with HIV (HIV+ groups).

Huber robust regression suggested that for PWH, a longer duration of living with HIV was associated with a greater BOLD response within the right ACC during 40¢ choices, *t*(44) = 3.8, *p* = 0.005 (Figure 3). No other clinical variables related to HIV nor a history of METH use were found to be associated with task-related BOLD response. Additionally, neither depressive symptoms nor trait impulsivity were associated with the BOLD response for participants with a history of METH use.

## 4. Discussion

The overall aim of the present study was to examine the impact of a history of METH use disorder on the neural substrates of risky decision-making in PWH using the Risky Gains Task [20,49,52] during functional neuroimaging. Despite similar choice behavior across groups, a general pattern emerged revealing that PWH and a history of METH use disorder (HIV+/METH+) had greater BOLD activation within the bilateral ACC and left caudate than either condition alone (i.e., HIV+/METH− and HIV−/METH+). Within the left ACC in particular, these differences may have been driven by anticipation of safe (20¢) choices. Moreover, a longer estimated duration of HIV infection was associated with greater right ACC BOLD activation to risky (40¢) choices for PWH regardless of METH use history. Taken together, these findings suggest that PWH and a history of METH use disorder may exhibit compensatory activation of regions associated with decision-making in the context of rewards [26,36].

Broadly consistent with previous findings using the RGT, PWH exhibited overall enhanced BOLD activation within the insula and putamen relative to PWoH [20]. A similar main of effect of METH use disorder history was seen within the bilateral insula where METH+ individuals demonstrated greater BOLD activation compared to METH−. Previous studies of the RGT similarly reported greater insular activation in METH+ individuals, particularly those at risk for relapse [55,59,60]. The insula has been heavily implicated in detecting and processing behaviorally salient stimuli [29,30,31,32] such as risky decisions as well as the affective integration of such events [29,33,34,35]. Greater insula activation has been associated with anticipating both monetary gain and loss [18,47,50,57], while also being sensitive to the level of risk involved [32,99,100,101]. Moreover, insular activity may reflect risk-taking urges [29,52,99] as greater activation has been observed in more impulsive individuals [102]. In this context, the overlapping but distinct pattern of BOLD activation within the insula for PWH and those with a history of METH use could reflect greater saliency and urge in anticipation of making monetary-related choices.

Contrary to our hypothesis, PWH and a history of METH use disorder exhibited overall increased activation within the ACC and caudate nucleus compared to either condition alone (Figure 1). Increased activation of the ACC has been associated with greater top-down implementation of cognitive control as well as detection of conflicting response options [31,38,39,40,41,42] while the caudate as part of the dorsal striatum is involved in goal-directed decision-making [36]. Both HIV and METH use have independently been associated with morphometric deficits of the ACC [103,104] and caudate [14,103,105]. The combined effects of both conditions could facilitate additional neuronal injury within the region [3,22,23,24,25,66], though no interactions on measures of morphometry have been detected to date [14,106]. Moreover, alterations of brain metabolites within prefrontal gray matter [66,107] and subcortical blood flow [108] have been observed in PWH and a history of METH use. Our sensitivity analyses additionally indicate that neural changes are independent of both medication status and active viral replications, suggesting potentially enduring alterations in the frontostriatal circuitry from the combined impact of HIV and METH [3,22,23,24,25]. These findings further highlight the potential for additive neural damage and decreased neuronal health from both HIV infection and METH use, which have been described in postmortem samples [109,110]. Taken together, HIV- and METH-related dysfunction of the underlying neuronal populations of the ACC and caudate could lead to decreased neural efficiency, necessitating compensatory activation [65] in order to maintain similar behavioral performance as observed in the present study.

Within the ACC more specifically, BOLD activation in anticipation of safe choices differentiated between groups (Figure 2). Consistent with previous studies, we found that both the HIV+/METH− and HIV−/METH+ groups exhibited hypoactivation of the ACC during anticipation of safe choices compared to seronegative, non-using controls [20,55,57]. Participants in these single condition groups additionally demonstrated similar within-group patterns of activation such that activation increased in response to the risky option with the highest potential gain or loss (80¢) relative to the safe choice. Individuals with a history of METH use may generally under-recruit cognitive control regions, such as the ACC, during decision-making [55,56,57,58]. Likewise, previous studies suggest that PWH may recruit compensatory ACC activation in the context of more difficult and ambiguous decisions [20,54,62,63,64]. Indeed, in our previous report using the RGT, we demonstrated that increased BOLD activation within the ACC, as well as the dorsal striatum among other regions, was related to lower nadir CD4 [20]. While we failed to replicate these findings, results of the robust regressions in the present study were complementary, demonstrating that greater ACC activation to risky choices was associated with a longer duration of HIV infection across PWH both with and without prior METH use (Figure 3). Together, these findings could suggest that HIV disease characteristics could mediate differences in neuronal activation and underlying neural damage [20,64,111].

Interestingly, participants in the dually affected HIV+/METH+ group demonstrated similar patterns of ACC activation as seronegative, non-using controls. Greater activation was observed during safe choices compared to both singly affected groups, and though only a strong trend, the HIV+/METH+ group demonstrated a greater response to safe choices compared to the riskiest option. In PWoH and no history of substance use, the BOLD response within the ACC has also been associated with loss aversion during risky decision-making paradigms, with activation decreasing as a function of risk [100,101]. In this context, it could suggest that PWH and a history of METH use dependence may display greater neural sensitivity to potential losses.

A small number of [100,101] studies have demonstrated that PWH and a history of METH use exhibit similar BOLD activation patterns as seronegative, non-using controls across a variety of tasks [17,19], which could suggest opposing effects of HIV and METH on the underlying neural circuitry. Relatedly, PWH and a history of cocaine use, which like METH modulates dopamine functioning [112], have demonstrated a similar pattern of enhanced BOLD activation that was most comparable to seronegative, non-using controls during risky decision-making and inhibitory control paradigms [113,114]. Activity within both the dorsal striatum [115] and ACC [116] is heavily regulated by dopamine, particularly in goal-directed behavior. Active METH use inhibits dopamine reuptake and causes release of dopamine from vesicles, resulting in increased extracellular dopamine [117]. HIV likewise induces dopaminergic dysfunction [118,119,120], with chronic, long-term exposure to HIV being associated with downregulation of dopamine functioning [121,122,123]. While few studies have examined the effects of HIV and METH on dopaminergic functioning in humans, animal studies suggest that HIV may sensitize the brain to METH exposure and reward [124,125]. In light of these considerations, it is possible that increased dopaminergic activity of METH use in the context of deficient dopamine functioning in HIV could help regulate neural functioning, giving rise to the findings in the present study where PWH and a history of METH use exhibit similar activation to seronegative, non-using controls.

## 5. Limitations

This study is not without its limitations. Given the cross-sectional nature of the study, it is not possible to know whether the findings are a consequence of METH use or presence of HIV infection or whether these findings are premorbid. More impulsive and reward-driven decision-making is known to be a risk factor for both substance use [126,127] and HIV [128], and could, thus, be reflected in the underlying neural substrates. METH use and METH use patterns can be impacted by the timing of HIV seroconversion [129] and could play a role in the deleterious effects on the brain. Likewise, the focus of this study was on the effects of METH use disorder in the context of HIV, but people with use of other substances, such as alcohol, cannabis, and nicotine, which are commonly used alongside METH, were not excluded. While we controlled for age in this study, the effects of both METH use and HIV may vary with age [24]. Future studies are needed to understand how these effects may vary across the lifespan, particularly as PWH are living longer thanks to the development of antiretroviral therapies [130]. Additionally, nearly all participants in this study were men, so the results may not generalize to women. Finally, given the self-driven nature of the RGT paradigm, participants were allowed to come up with their own strategy which resulted in many participants only choosing the safe option which precluded them from being part of the fMRI analysis and led to a smaller final sample size.

## 6. Conclusions

The present study examined the independent and combined effects of a history of METH use and HIV on brain activation during risky decision-making. We found that both METH and HIV were independently associated with increased insular activation across both safe and risky choices. Within the bilateral ACC and caudate nucleus, the overall BOLD response was modulated as a function of both HIV and METH use history such that HIV+/METH+ individuals exhibited a pattern of increased activation similar to seronegative, non-using controls. These findings suggest changes to underlying neural functioning due to HIV and METH exposure.

## Figures and Tables

**Table 1:** Demographic and clinical characteristics of participants.

					F/χ^2^		
Characteristic	HIV−/METH− *n* = 23	HIV−/METH+ *n* = 21	HIV+/METH− *n* = 20	HIV+/METH+ *n* = 24	HIV	METH	HIV × METH
Age	44.6 (±11.7)	41.4 (±11.0)	41.2 (±9.3)	41.3 (±8.9)	0.6	0.5	0.6
Education (years)	14.3 (±2.1)	13.0 (±2.4)	14.0 (±2.5)	14.2 (±1.9)	0.7	1.5	2.8
Ethnicity					2.3	0.3	1.4
White	13 (57%)	8 (38%)	12 (60%)	16 (67%)			
Hispanic	5 (22%)	8 (38%)	4 (20%)	6 (25%)			
Black	5 (22%)	4 (19%)	4 (20%)	2 (8.3%)			
Asian	0 (0%)	1 (4.8%)	0 (0%)	0 (0%)			
Sex (% male)	18 (78%)	15 (71%)	18 (90%)	24 (100%)	10.0 **	2.2	3.5 †
Scanned on 3T GE Discovery MR 750	18 (78%)	14 (67%)	18 (90%)	20 (83%)	2.7	1.1	0.0
Wide Range Achievement Test—4 (Standard Score)	106 (±15)	97 (±13)	104 (±13)	107 (±13)	2.0	1.1	3.3
Global Deficit Score	0.25 (±0.29)	0.48 (±0.40)	0.26 (±0.32)	0.33 (±0.32)	1.0	4.5 *	1.2
Beck Depression Inventory—II (at time of MRI)	3.7 (±5.0)	7.9 (±9.0)	5.6 (±5.6)	10.5 (±10.6)	1.7	7.0 **	0.0
Barratt Impulsiveness Scale (Total Score)	57.3 (±10.4)	72.8 (±13.2)	62.3 (±12.3)	69.2 (±14.6)	0.1	16.2 ***	2.3
Kalichman Sensation Seeking Scale							
Non-Sexual Sensation-Seeking	2.11 (±0.58)	2.29 (±0.85)	1.94 (±0.50)	2.17 (±0.64)	1.0	2.0	0.0
Sexual Sensation-Seeking	1.87 (±0.50)	2.48 (±0.78)	2.29 (±0.59)	2.59 (±0.67)	3.7 †	10.7 **	1.2
Sexual Compulsivity	1.23 (±0.55)	1.74 (±0.83)	1.50 (±0.52)	1.67 (±0.71)	0.4	5.5 *	1.4
Methamphetamine Characteristics							
Age of First Use		24.3 (±10.5)		25.3 (±6.9)	0.1		
Total Days Used		2679 (±1877)		1923 (±2004)	1.6		
Days Since Last Use		145 (±186)		157 (±165)	0.1		
Total Quantity (g)		3979 (±4595)		1860 (±2973)	3.4		
Use Density		1.26 (±1.17)		0.85 (±0.62)	2.2		
Primary Route of Use (smoking)		13 (65%)		12 (50%)	2.5		
HIV Characteristics							
Duration of Infection (years)			11.0 (±9.0)	8.2 (±6.9)		1.4	
Virally Suppressed (≤50 copies/mL)			15 (75%)	15 (63%)		0.8	
Nadir CD4 Count			210.8 (±176.2)	298.3 (±224.2)		2.0	
Current CD4 Count			537.5 (±336.0)	589.0 (±255.6)		0.3	
Current Antiretroviral Use			17 (85%)	21 (88%)		0.1	
Current Antiretroviral Regimen						0.6	
NRTI/II			7 (41%)	9 (43%)			
NNRTI/NRTI			4 (24%)	7 (33%)			
PI/NRTI			3 (18%)	4 (19%)			
NNRTI/II			1 (5.9%	0 (0%)			
3-class			2 (12%)	1 (4.8%)			
Cumulative Duration of Antiretroviral Treatment (months)			84.6 (±82.1)	62.2 (±57.2)		1.1	

Note: Mean (±SD); *n*(%); *** *p* < 0.001; ** *p* < 0.01; * *p* < 0.05; † < 0.10; NRTI: nucleoside reverse transcriptase inhibitor; II: integrase inhibitor; NNRTI: non-nucleoside reverse transcriptase inhibitor; PI: protease inhibitor.

**Table 2:** Regions of interest with significant BOLD activation for HIV × METH × Choice (20¢, 40¢, 80¢) linear mixed-effects analysis.

Structure	Volume (μL)	X	Y	Z	F-Value	Post Hoc Comparisons		
						Contrast	t-Ratio	ES
Main Effect of Choice								
Left Caudate Nucleus	432	−11	9	8	28.2 ***	20¢ > 40¢ 20¢ > 80¢	−5.5 *** −7.2 ***	−0.28 −0.36
Left Insula Lobe	1782	−33	20	−1	37.8 ***	20¢ > 40¢ 20¢ > 80¢ 40¢ > 80¢	−5.2 *** −8.5 *** −3.3 **	−0.29 −0.47 −0.18
Right Insula Lobe	1242	34	21	2	43.7 ***	20¢ > 40¢ 20¢ > 80¢ 40¢ > 80¢	−6.2 *** −9.1 *** −2.9 **	−0.35 −0.51 −0.16
Main Effect of HIV								
Left Insula Lobe	405	−40	−12	−1	7.0 **	HIV− > HIV+	−2.6 **	−0.22
Right Putamen	1215	38	−10	0	19.5 ***	HIV− > HIV+	−4.4 ***	−0.32
Main Effect of METH								
Left Insula Lobe	945 513	−35 −40	12 −10	−11 2	8.8 ** 6.3 *	METH− > METH+ METH− > METH+	−3.0 ** −2.5 *	−0.26 −0.19
Right Insula Lobe	621	36	13	−14	5.6 *	METH− > METH+	−2.4 *	−0.28
HIV × METH								
Right Anterior Cingulate Cortex	945	6	18	24	10.5 **	METH+: HIV− > HIV+ HIV+: METH− > METH+	−3.4 ** −3.0 **	−0.35 −0.31
Left Anterior Cingulate Cortex	837	1	30	12	7.7 **	METH+: HIV− > HIV+ HIV+: METH− > METH+	−2.3 * −2.9 *	−0.32 −0.40
Left Caudate Nucleus	567	−12	3	18	8.0 **	METH+: HIV− > HIV+ HIV+: METH− > METH+	−3.2 ** −2.4 *	−0.40 −0.30
HIV × METH × Choice								
Left Anterior Cingulate Cortex	621	1	30	11	14.4 ***	20¢, METH−: HIV− > HIV+ 20¢, METH+: HIV− > HIV+ 20¢, HIV−: METH− > METH+ 20¢, HIV+: METH− > METH+ HIV−/METH−: 20¢ > 80¢ HIV−/METH+: 20¢ > 80¢ HIV+/METH−: 20¢ > 80¢ HIV+/METH+: 20¢ > 80¢	3.3 * −3.7 ** 2.7 * −4.3 *** 3.0 * −2.4 † −2.9 * 2.3 †	0.58 −0.63 0.46 −0.74 0.33 −0.28 −0.34 0.25

*** *p* < 0.001; ** *p* < 0.01; * *p* < 0.05; † < 0.07.

## Data Availability

Data are maintained in secure repositories at the HIV Neurobehavioral Research Program, University of California San Diego, and are available upon request via hnrpresource@ucsd.edu).

## Supplementary Materials

The following supporting information can be downloaded at https://www.mdpi.com/article/10.3390/v18030369/s1, Supplementary Results: Excluded participant demographics; behavioral analyses with all participants; Table S1: Whole brain clusters with significant BOLD activation for HIV × METH × Choice (20¢, 40¢, 80¢) linear mixed-effects analysis; Table S2: Demographic and clinical characteristics of participants excluded due to choice behavior on the Risky Gains Task; Table S3: Regions of interest with significant BOLD activation for HIV × METH × Choice (20¢, 40¢, 80¢) linear mixed-effects analysis excluding people with HIV who were not on an antiretroviral regimen; Table S4. Regions of interest with significant BOLD activation for HIV × METH × Choice (20¢, 40¢, 80¢) linear mixed-effects analysis excluding people with HIV who were not virally suppressed; Figure S1: Choice behavior from the Risky Gains Task; Figure S2: Bar plots showing significant main effect of Choice; Figure S3: Bar plots showing significant main effect of HIV within two clusters; Figure S4: Bar plots showing significant main effect of METH within two clusters.
