# Supplementary material for: A History of Methamphetamine Use Disorder in People with HIV Is Associated with Altered Functional Response to Risky Choice
Source: Viruses. 2026 Mar 17;18(3):369. doi: 10.3390/v18030369 (PMC13030450; doi:10.3390/v18030369)
Supplement: Supplementary file 1 [file viruses-18-00369-s001.zip › viruses-4136421-supplementary.pdf]

Article

A History of Methamphetamine Use Disorder in People with HIV is Associated with Altered Functional Response to Risky Choice

Joseph P. Happer[1]*, Susan F. Tapert[1], Igor Grant[1], Amanda Bischoff-Grethe[1], and on behalf of the Translational Methamphetamine AIDS Research Center (TMARC) Group

Supplementary Material

Results

Excluded Participant Demographics

Participants excluded from the analysis due to choice behavior were analyzed separately to ensure that they were not systematically different in terms of their demographic and clinical characteristics (see Table S2). Excluded participants did not differ on age or WRAT-4 standard scores. However, there was a main effect of METH use history on years of education, $F(1,48) = 22.7$, $p < 0.0001$, $\eta_p^2 = 0.32$, with participants who are METH+ attaining few years of education. However, given there were no difference in WRAT-4 scores, $F(1,48) = 2.2$, $p = 0.14$, $\eta_p^2 = 0.04$, suggests that though METH+ individuals attained fewer years of education, estimates of premorid functioning and general intelligence were comparable across groups. There was a main effect of HIV with excluded PWoH being predominantly white compared to PWH who were more ethnically diverse, $\chi^2 = 4.6$, $p = 0.03$, Cramer's $V = 0.11$. Finally, there was an additional main effect of METH use history on KS4 non-sexual sensation seeking, $F(1,48) = 6.0$, $p = 0.02$, $\eta_p^2 = 0.11$, with METH+ individuals having higher scores, which was not evident in the primary analysis.

Like those included in the primary analysis, excluded individuals did not differ on METH-related or HIV-related clinical variables.

Behavioral Analysis

To ensure that our exclusionary criteria based on choice behavior did not bias our findings, we conducted a sensitivity analysis by analyzing the behavioral data with all participants included. A main effect of Choice was observed, $F(2,272) = 319.9$, $p < .0001$, $\eta_p^2 = 0.7$. Post-hoc *t*-tests indicated that participants primarily made safe (20¢) choices, which were selected more than both risky choices (40¢: $t(272) = 20.4$, $p < 0.0001$, ES = 2.5; 80¢: $t(272) = 23.1$, $p < 0.0001$, ES = 2.8). Participants similarly chose the less risky option (40¢) more than the riskiest (80¢) choice $t(272) = 2.7$, $p = 0.008$, ES = 0.3. No differences in choice behavior were detected based on METH or HIV status, nor as an interaction (all *p*s > 0.2).

Supplementary Figures and Tables

Table S1. Whole brain clusters with significant BOLD activation for HIV x METH x Choice (20¢, 40¢, 80¢) linear mixed effects analysis.

Structure	Volume (μL)	X	Y	Z	F-value	Post Hoc Comparisons		
						Contrast	t-Ratio	ES
Main Effect of Choice								
Right Fusiform Gyrus	648	26	−49	−13	22.1***	20¢ > 40¢	−4.6***	−0.27
						20¢ > 80¢	−6.5***	−0.38
Right Inferior Frontal Gyrus (pars triangularis)	945	40	18	23	57.2***	20¢ > 40¢	−6.6***	−0.34
						20¢ > 80¢	−10.6***	−0.55

Structure	Volume (μL)	X	Y	Z	F-value	Post Hoc Comparisons		
						Contrast	t-Ratio	ES
						40¢ > 80¢	−4.0***	−0.21
Right Inferior Parietal Lobule	594	33	−47	50	63.4***	20¢ > 40¢	−8.7***	−0.38
						20¢ > 80¢	−10.6***	−0.47
Right Middle Occipital Gyrus	810	31	−66	32	65.2***	20¢ > 40¢	−8.1***	−0.38
						20¢ > 80¢	−11.0***	−0.52
						40¢ > 80¢	−2.9**	−0.14
Left Precentral Gyrus	567	−25	−13	54	26.7***	20¢ > 40¢	−6.3***	−0.24
						20¢ > 80¢	−6.4***	−0.24
Right Precuneus	702	6	−52	50	37.3***	20¢ > 40¢	−6.5***	−0.31
						20¢ > 80¢	−8.2***	−0.39
Right Superior Frontal Gyrus	486	30	−6	58	22.7***	20¢ > 40¢	−5.6***	−0.28
						20¢ > 80¢	−6.1***	−0.30
Left Superior Parietal Lobule	5670	−28	−61	46	72.1***	20¢ > 40¢	−8.8***	−0.35
						20¢ > 80¢	−11.5***	−0.45
						40¢ > 80¢	−2.7**	−0.10
Main Effect of HIV								
Right Lingual Gyrus	459	16	−48	2	4.9*	HIV− > HIV+	−2.2*	−0.22
Left Lingual Gyrus	405	−19	−60	−10	15.8***	HIV− > HIV+	−4.0***	−0.35
Main Effect of METH								
Left Inferior Frontal Gyrus (pars orbitalis)	1890	−39	30	−13	7.3**	METH− > METH+	−2.7**	−0.34
Left Medial Temporal Pole	783	−39	17	−35	8.1**	METH− > METH+	−2.8**	−0.35
Left Middle Frontal Gyrus	486	−28	17	44	12.5***	METH− > METH+	3.5***	0.24
Left Middle Orbital Gyrus	486	−28	49	−9	10.5**	METH− > METH+	−3.2**	−0.43
Left Olfactory cortex	783	−22	5	−15	12.9***	METH− > METH+	−3.6***	−0.43
Left Superior Frontal Gyrus	540	−14	44	37	6.5*	METH− > METH+	2.5*	0.22
Left Superior Medial Gyrus	567	−8	41	46	6.0*	METH− > METH+	2.5*	0.24
Right Inferior Frontal Gyrus (pars orbitalis)	540	44	41	−10	5.6*	METH− > METH+	−2.4*	−0.27
Right Inferior Frontal Gyrus (pars triangularis)	648	49	33	0	15.7***	METH− > METH+	−4.0***	−0.45
Right Insula Lobe	378	36	19	−14	7.2**	METH− > METH+	−2.7**	−0.34
Right Superior Medial Gyrus	567	8	36	51	12.0***	METH− > METH+	3.5***	0.30
METH x Choice								
Left Inferior Frontal Gyrus (pars orbitalis)	864	−46	41	−15	2.9†	METH+: 20¢ > 40¢	2.8*	0.28
						METH+: 20¢ > 80¢	2.8*	0.29
HIV x METH								
Left Precentral Gyrus	378	−42	1	45	18.3***	METH+: HIV− > HIV+	−4.5***	−0.43
						HIV−: METH− > METH+	4.1***	0.40

*** $p < 0.001$; ** $p < 0.01$; * $p < 0.05$; † < 0.07

Table S2. Demographic and clinical characteristics of participants excluded due to choice behavior on the Risky Gains Task

Characteristic	HIV−/METH−	HIV−/METH+	HIV+/METH−	HIV+/METH+	F/χ		
	n = 18	*n* = 10	*n* = 14	*n* = 10	HIV	METH	HIV x METH
Age	47.5 (±15.7)	40.2 (±11.6)	46.7 (±10.8)	41.9 (±8.1)	0.0	2.9	0.1
Education (years)	14.8 (±2.2)	10.9 (±1.6)	14.3 (±2.0)	12.7 (±2.0)	1.3	22.7***	4.0†
Ethnicity					4.6*	0.1	0.0
White	12 (67%)	7 (70%)	5 (36%)	4 (40%)			
Hispanic	4 (22%)	1 (10%)	4 (29%)	5 (50%)			
Black	2 (11%)	2 (20%)	3 (21%)	1 (10%)			
Asian	0 (0%)	0 (0%)	0 (0%)	0 (0%)			
Other	0 (0%)	0 (0%)	2 (14%)	0 (0%)			
Sex (% male)	8 (44%)	9 (90%)	13 (93%)	10 (100%)	4.8*	3.6†	0.1
Scanned on 3T GE Discovery MR 750	14 (78%)	6 (60%)	13 (93%)	8 (80%)	2.4	1.8	0.0
Wide Range Achievement Test − 4 (Standard Score)	107 (±13)	99 (±12)	99 (±8)	97 (±8)	2.7	2.2	1.1
Global Deficit Score	0.35 (±0.36)	0.33 (±0.31)	0.56 (±0.49)	0.43 (±0.32)	2.0	0.5	0.3
Beck Depression Inventory II (at time of MRI)	2.0 (±2.8)	10.8 (±8.5)	6.0 (±9.8)	13.4 (±12.4)	1.6	9.7**	0.1
Barratt Impulsiveness Scale (Total Score)	57.6 (±13.5)	71.2 (±16.8)	56.4 (±10.5)	67.6 (±10.6)	0.4	11.0**	0.1
Kalichman Sensation Seeking Scale							
Non-Sexual Sensation Seeking	1.75 (±0.62)	2.18 (±0.28)	1.77 (±0.50)	2.10 (±0.57)	0.0	6.0*	0.1
Sexual Sensation Seeking	1.62 (±0.51)	2.52 (±0.60)	2.19 (±0.71)	2.47 (±0.67)	2.1	10.7**	3.0
Sexual Compulsivity	1.14 (±0.28)	1.69 (±0.80)	1.28 (±0.37)	1.44 (±0.75)	0.1	5.2*	1.5
Methamphetamine Characteristics							
Age of First Use		23.4 (±10.4)		23.8 (±9.9)	0.0		
Total Days Used		3,302 (±2,256)		3,182 (±2,988)	0.0		
Days Since Last Use		276 (±678)		153 (±136)	0.3		
Total Quantity (g)		3,519 (±6,131)		5,097 (±8,101)	0.2		
Use Density		1.03 (±0.98)		1.37 (±0.88)	0.6		
Primary Route of Use (smoking)		8 (80%)		5 (50%)	1.4		
HIV Characteristics							
Duration of Infection (years)			11.2 (±10.5)	11.2 (±7.2)		0.0	
Virally Suppressed (<= 50 copies/mL)			10 (83%)	6 (67%)		0.8	
Nadir CD4 Count			302.6 (±206.4)	200.7 (±117.8)		2.0	
Current CD4 Count			600.1 (±283.9)	546.8 (±196.2)		0.2	
Current ART Use			13 (93%)	8 (80%)		0.9	

**Characteristic**	**HIV−/METH−** *n* = 18	**HIV−/METH+** *n* = 10	**HIV+/METH−** *n* = 14	**HIV+/METH+** *n* = 10	**F/χ**		
					**HIV**	**METH**	**HIV x METH**
Current ART Regimen						0.0	
NRTI/II			2 (15%)	2 (25%)			
NNRTI/NRTI			5 (38%)	2 (25%)			
PI/NRTI			3 (23%)	3 (38%)			
3-class			2 (15%)	1 (13%)			
PI/II			1 (7.7%)	0 (0%)			
Cumulative Duration of Antiretroviral Treatment (months)			90.1 (±101.6)	83.0 (±70.6)		0.0	

Mean (±SD); n(%); ***p < 0.001; **p < 0.01; *p < 0.05; † < 0.10;

NRTI: nucleoside reverse transcriptase inhibitor; II: integrase inhibitor; NNRTI: non-nucleoside reverse transcriptase inhibitor; PI: protease inhibitor

Table S3. Regions of interest with significant BOLD activation for HIV x METH x Choice (20¢, 40¢, 80¢) linear mixed effects analysis excluding people with HIV who were not on an antiretroviral regimen.

Structure	Volume (μL)	X	Y	Z	F-value	Post Hoc Comparisons		
						Contrast	t-Ratio	ES
Main Effect of Choice								
Left Caudate Nucleus	432	−11	9	8	25.1***	20¢ > 40¢	−5.3***	−0.28
						20¢ > 80¢	−6.7***	−0.35
Left Insula Lobe	1836	−33	20	−1	30.5***	20¢ > 40¢	−4.8***	−0.28
						20¢ > 80¢	−7.7***	−0.44
						40¢ > 80¢	−2.9**	−0.17
Right Insula Lobe	1242	34	21	2	40.4***	20¢ > 40¢	−6.1***	−0.35
						20¢ > 80¢	−8.8***	−0.50
						40¢ > 80¢	−2.6**	−0.15
Main Effect of HIV								
Right Insula Lobe	675	40	−4	−8	13.4***	HIV− > HIV+	−3.7***	−0.31
Main Effect of METH								
Left Insula Lobe	810	−35	12	−10	8.1**	METH− > METH+	−2.9**	−0.26
	486	−39	10	−3	7.7**	METH− > METH+	−2.8**	−0.22
Right Insula Lobe	513	35	12	−15	3.1†	METH− > METH+	−1.8†	−0.22
HIV x METH								
Left Anterior Cingulate Cortex	729	1	29	11	7.7**	METH+: HIV− > HIV+	−2.3*	−0.34
						HIV+: METH− > METH+	−2.7*	−0.42
Left Caudate Nucleus	540	−12	2	18	9.0**	METH+: HIV− > HIV+	−3.2**	−0.42
						HIV+: METH− > METH+	−2.6*	−0.36
Right Anterior Cingulate Cortex	891	5	18	24	9.0**	METH+: HIV− > HIV+	−3.0*	−0.34
						HIV+: METH− > METH+	−2.7*	−0.32
HIV x METH x Choice								
Left Anterior Cingulate Cortex	540	1	30	12	13.2***	20¢, HIV−: METH− >	2.7*	0.48
						METH+	−4.0**	−0.75
						20¢, HIV+: METH− >	2.9*	0.54
						METH+	−3.9**	−0.69
						20¢, METH−: HIV− > HIV+	2.8*	0.30
						20¢, METH+: HIV− > HIV+	−2.3†	−0.26
						HIV−/METH−: 20¢ > 80¢	−2.7*	−0.30

	HIV−/METH+: 20¢ > 40¢	−2.2†	−0.27
	HIV−/METH+: 20¢ > 80¢	2.2†	0.25
	HIV+/METH−: 20¢ > 80¢	2.4†	0.28
	HIV+/METH+: 20¢ > 40¢		
	HIV+/METH+: 20¢ > 80¢		

***$p < 0.001$; **$p < 0.01$; *$p < 0.05$; † < 0.10

Table S4. Regions of interest with significant BOLD activation for HIV x METH x Choice (20¢, 40¢, 80¢) linear mixed effects analysis excluding people with HIV who were not virally suppressed.

Structure	Volume (µL)	X	Y	Z	F-value	Post Hoc Comparisons		
						Contrast	t-Ratio	ES
Main Effect of Choice								
Left Insula Lobe	1728	−33	21	−2	32.9***	20¢ > 40¢	−5.3***	−0.33
						20¢ > 80¢	−8.0***	−0.50
						40¢ > 80¢	−2.7**	−0.17
Right Insula Lobe	1242	34	21	2	44.6***	20¢ > 40¢	−6.6***	−0.41
						20¢ > 80¢	−9.2***	−0.56
						40¢ > 80¢	−2.6*	−0.16
Main Effect of METH								
Left Insula Lobe	864	−36	12	−10	5.5*	METH− > METH+	−2.3*	−0.24
	459	−39	−10	−3	4.6*	METH− > METH+	−2.2*	−0.18
Right Insula Lobe	513	35	12	−15	1.8	METH− > METH+	−1.3	−0.18
HIV x METH								
Right Anterior Cingulate Cortex	891	5	18	24	9.0**	METH+: HIV− > HIV+	−3.1**	−0.39
						HIV−: METH− > METH+	1.5	0.17
						HIV+: METH− > METH+	−2.7*	−0.35
HIV x METH x Choice								
Right Anterior Cingulate Cortex	540	5	20	23	12.1***	20¢, HIV−: METH− > METH+	3.1*	0.43
						20¢, HIV+: METH− > METH+	−4.0**	−0.67
						20¢, METH−: HIV− > HIV+	2.8*	0.43
						20¢, METH+: HIV− > HIV+	−4.2**	−0.66
						HIV−/METH+: 20¢ > 40¢	−3.0*	−0.31
						HIV−/METH+: 20¢ > 80¢	−3.9**	−0.40
						HIV+/METH−: 20¢ > 40¢	−3.3**	−0.39
						HIV+/METH−: 20¢ > 80¢	−3.8**	−0.45

***p < 0.001; **p < 0.01; *p < 0.05; † < 0.10

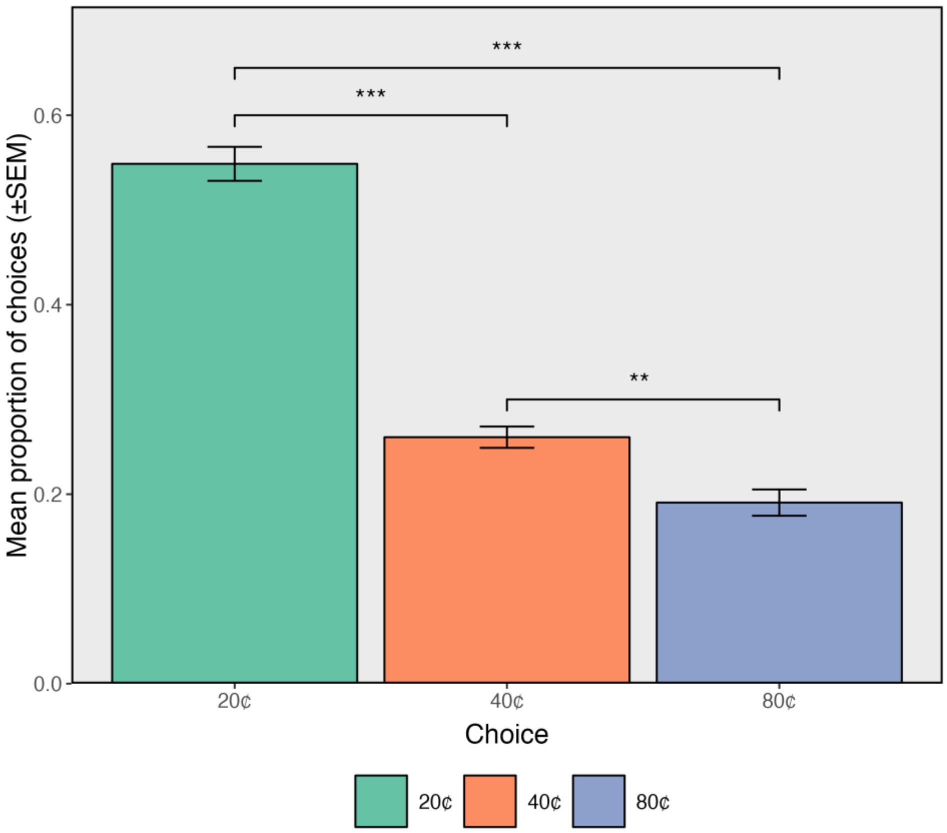

Figure S1. Choice behavior from the Risky Gains Task. Overall, participants made more 20¢ choices compared to 40¢ and 80¢ choices. Similarly, participants made more 40¢ choices compared to 80¢. No differences in choice behavior were detected based on HIV or METH group status. *** $p < 0.001$; ** $p < 0.01$

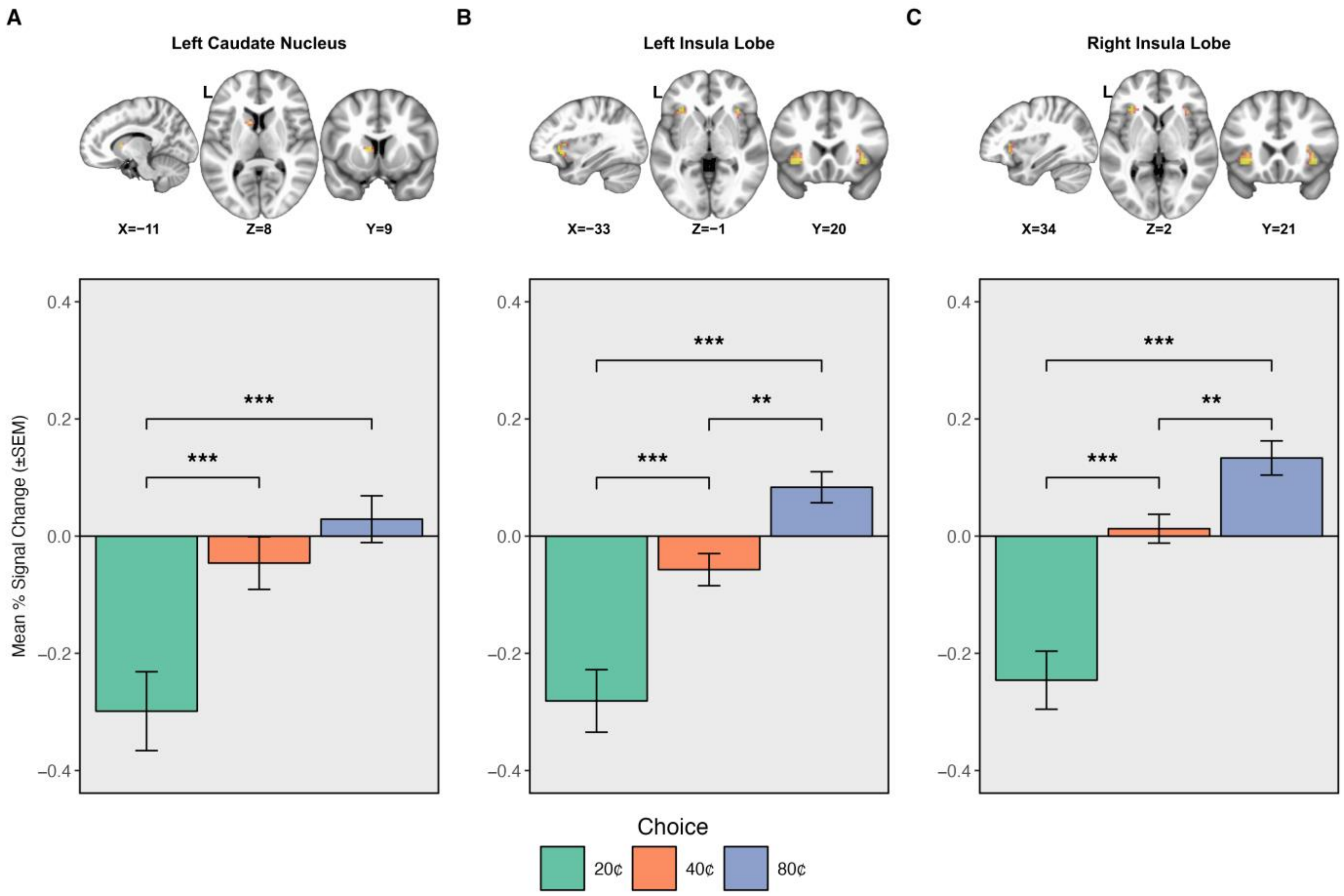

Figure S2. Bar plots showing significant main effect of Choice. Within the left caudate nucleus (A) left insula (B), and right insula (C) participants exhibited overall greater BOLD response to 40¢ and 80¢ choices compared to 20¢ choices. Within the left and right insula, there was a similar pattern of greater BOLD response to 80¢ choices relative to 40¢ choices. *** $p < 0.001$; ** $p < 0.01$

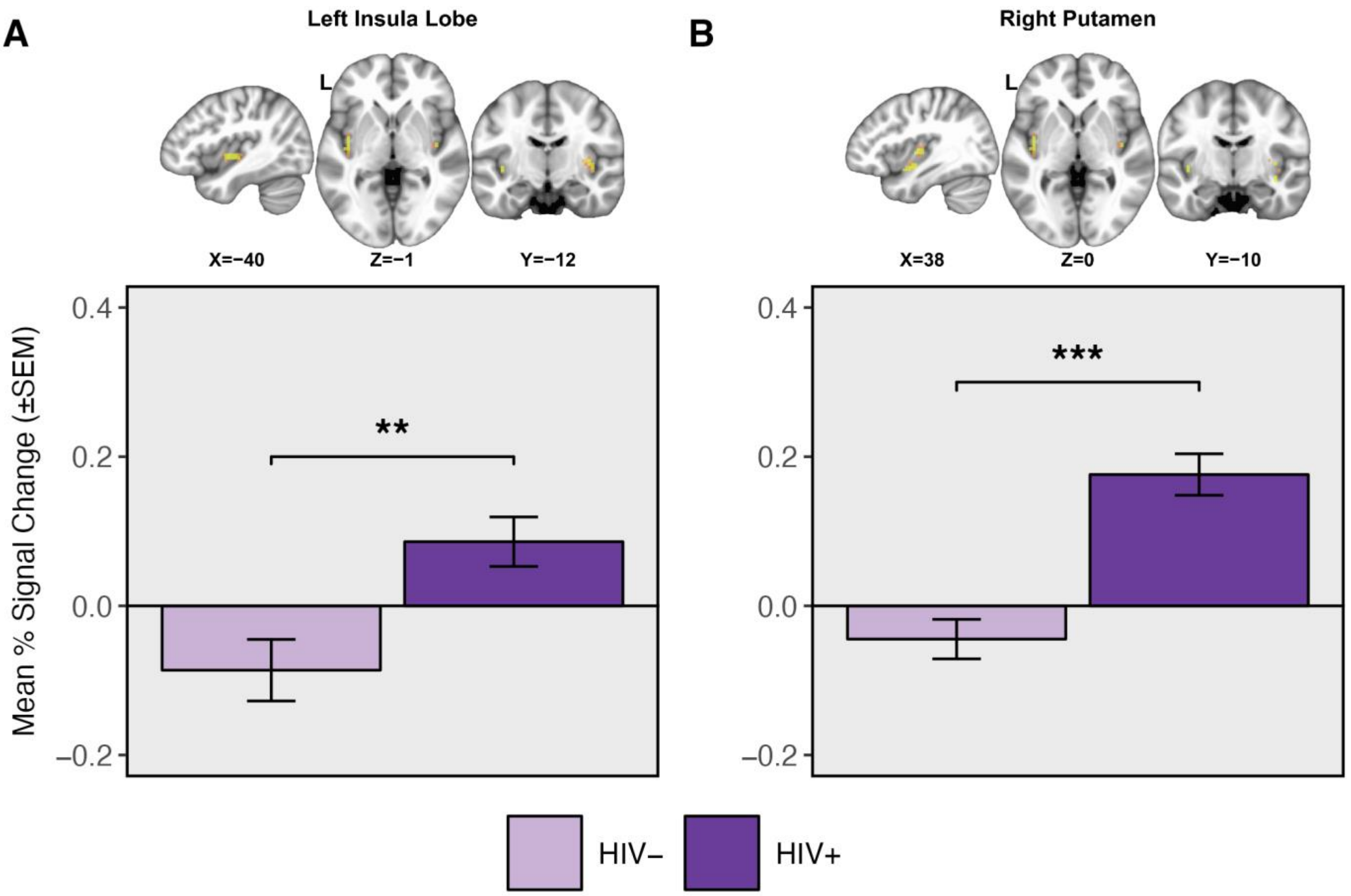

Figure S3. Bar plots showing significant main effect of HIV within two clusters. Within the right putamen (A) and left insula (B) people with HIV (HIV+) exhibited overall greater BOLD than those without HIV (HIV−). *** p < 0.001; ** p < 0.01

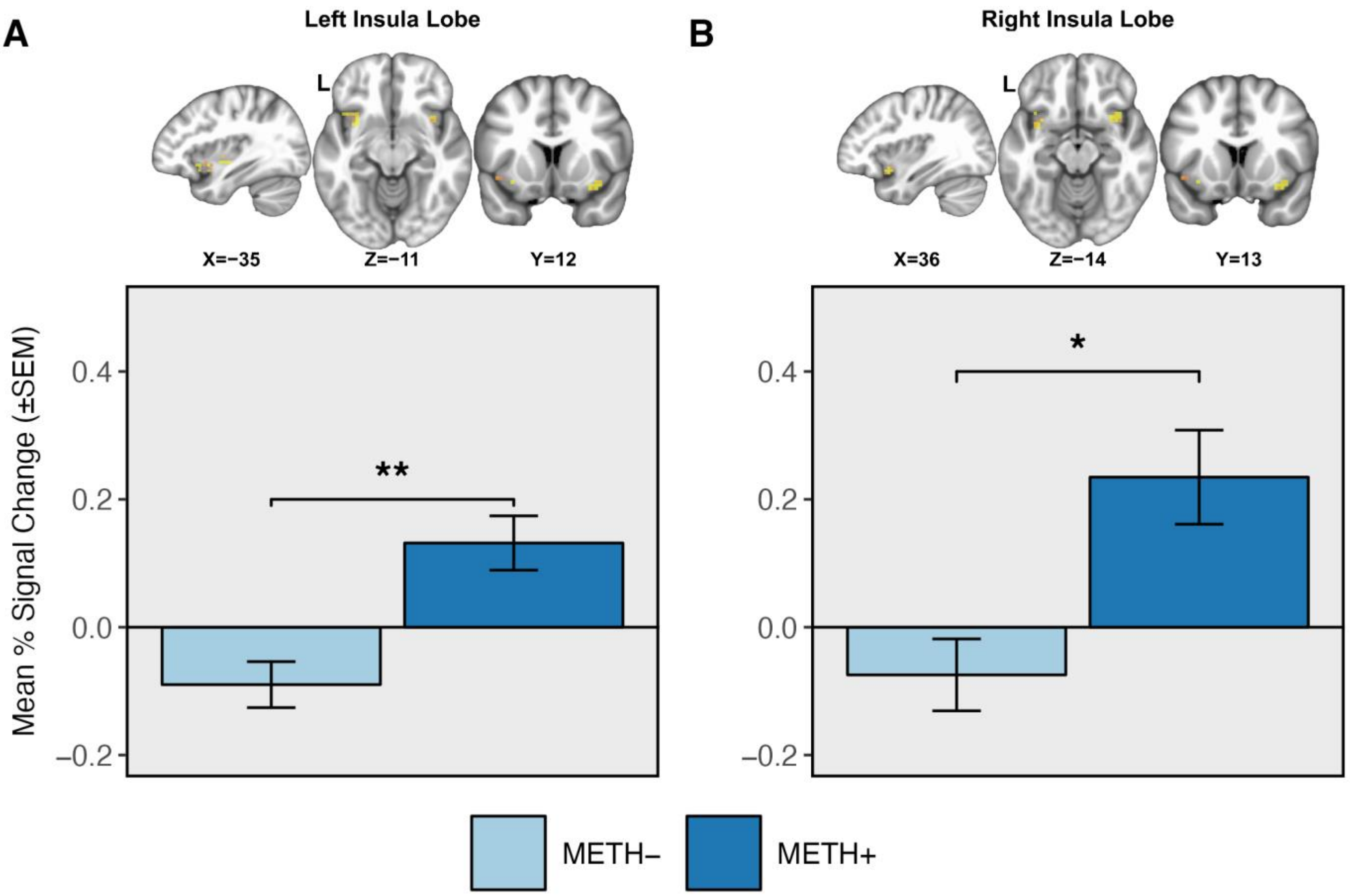

Figure S4. Bar plots showing significant main effect of METH within two clusters. Within the left (A) and right (B) insula, participants with a history of METH use disorder (METH+) exhibited overall greater BOLD response than those without a METH use history (METH−). ** $p < 0.01$; * $p < 0.05$
